# A novel bead-based assay to detect specific antibody responses against *Toxoplasma gondii *and *Trichinella spiralis *simultaneously in sera of experimentally infected swine

**DOI:** 10.1186/1746-6148-8-36

**Published:** 2012-03-28

**Authors:** Gertie CAM Bokken, Aldert A Bergwerff, Frans van Knapen

**Affiliations:** 1 Institute for Risk Assessment Sciences (IRAS), Division of Veterinary Public Health, Faculty of Veterinary Medicine, Utrecht University, Yalelaan 2, 3584 CM Utrecht, The Netherlands; 2 Department of Veterinary Public Health and Food Safety, Faculty of Veterinary Medicine, Ghent University, Salisburylaan 133, 9820 Merelbeke, Belgium

## Abstract

**Background:**

A novel, bead-based flow cytometric assay was developed for simultaneous determination of antibody responses against *Toxoplasma gondii *and *Trichinella spiralis *in pig serum. This high throughput screening assay could be an alternative for well known indirect tests like ELISA. One of the advantages of a bead-based assay over ELISA is the possibility to determine multiple specific antibody responses per single sample run facilitated by a series of antigens coupled to identifiable bead-levels. Furthermore, inclusion of a non-coupled bead-level in the same run facilitates the determination of, and correction for non-specific binding. The performance of this bead-based assay was compared to one *T. spiralis *and three *T. gondii *ELISAs. For this purpose, sera from *T. gondii *and *T. spiralis *experimentally infected pigs were used. With the experimental infection status as gold standard, the area under the curve, Youden Index, sensitivity and specificity were determined through receiver operator curve analysis. Marginal homogeneity and inter-rater agreement between bead-based assay and ELISAs were evaluated using McNemar's Test and Cohen's kappa, respectively.

**Results:**

Results indicated that the areas under the curve of the bead-based assay were 0.911 and 0.885 for *T. gondii *and *T. spiralis*, respectively, while that of the *T. gondii *ELISAs ranged between 0.837 and 0.930 and the *T. spiralis *ELISA was 0.879. Bead-based *T. gondii *assay had a sensitivity of 86% and specificity of 96%, while the ELISAs ranged between 64-84% and 93-99%, respectively. The bead-based *T. spiralis *assay had a sensitivity of 68% and specificity of 100% while the ELISA scored 72% and 95%, respectively. Marginal homogeneity was found between the *T. gondii *bead-based test and one of the *T. gondii *ELISAs. Moreover, in this test combination and between *T. spiralis *bead-based assay and respective ELISA, an excellent inter-rater agreement was found. When results of samples before expected seroconversion were removed from evaluation, notably higher test specifications were found.

**Conclusions:**

This new bead-based test, which detects *T. gondii *and *T. spiralis *antibodies simultaneously within each sample, can replace two indirect tests for the determination of respective antibodies separately, while performing equally well or better.

## Background

*Trichinella spiralis *and *Toxoplasma gondii *are well known zoonoses which can pass from pigs to humans by consumption of raw or undercooked infected pork. In humans, most cases of *T. gondii *and *T. spiralis *infections go undetected; however, some cases can lead to mild disease. Other cases of trichinellosis can be very severe and may lead to myocarditis, encephalitis or pneumonia. Post natal acquired toxoplasmosis can incidentally lead to encephalitis and necrotizing retinochoroiditis, while congenital transmitted toxoplasmosis can lead to mental retardation, convulsions, spasticity, cerebral palsy, deafness and severely impaired vision in the offspring. In rare occasions, both these infections can lead to death.

These days, in Europe, trichinellosis is rarely reported in association with the consumption of pork from conventionally raised pigs [1]. An EU regulation [2] directs inspection of *T. spiralis *in each pig carcass at slaughter by direct parasitological methods. This regulation also states that serological tests may be implemented as a supplement for monitoring purposes.

Unlike *T. spiralis*, no such regulations exist for *T. gondii*, although the prevalence of this parasite in pigs is higher and health consequences of toxoplasmosis can be, like those of trichinellosis, rather serious. For example, in a Dutch survey in 2004, *T. gondii *infection was found in 2.6% of the studied pigs [3], while in that year none of the over 13 million slaughtered pigs were found *T. spiralis *positive [4].

Consumption of raw or undercooked *T. gondii *infected pork may cause toxoplasmosis in humans. Obviously, determination of the *T. gondii *status of the meat producing pigs, subsequently followed by precautionary methods, like freezing of pork to kill the parasite or altogether removal of this meat from the food chain, could contribute to fewer infections in humans. A Dutch study which assessed the epidemiology and impact of, amongst others, *T. gondii *infections in humans indicated that this parasite is one of the major contributors of disease through zoonotic transmission [5]. Similarly, an American study indicated that *T. gondii *in pork ranked second on the list of the zoonotic micro-organisms with the greatest impact on annual disease burden in that country [6] and was only surpassed by *Campylobacter *in chicken. In a scientifically opinion to the European Food Safety Authority (EFSA) it is recommended that standardized methods should be used on *T. gondii *pre-harvest monitoring of, amongst others, pigs [7].

Like *T. spiralis*, the *T. gondii *infection status of animals can be examined by serological tests in order to produce *T. gondii *controlled pork. Testing serum samples of finisher pigs requires an automated and easy to perform test method with a high sensitivity (Se) and specificity (Sp). Enzyme-linked immunosorbent assays (ELISAs) are such test methods which are commonly used.

Bead-based assays (BBA) are a new dimension in the determination of specific antibody responses. The test is performed on beads which are available in different sizes and levels. During flow cytometric analysis individual beads are distinguishable by size and intrinsic fluorescence intensity level. The bead surface is carboxylate modified, which allows covalent coupling of protein. The great advantage of these tests over ELISA is the possibility of simultaneous detection of specific responses against multiple antigens per single serum sample. More specifically, by individual coupling of antigens to specific bead levels, and combination of these bead levels per test sample, a multitude of specific responses can be determined simultaneously per sample. Furthermore, by the use of a non-coupled bead, non-specific binding (NSB) can be monitored and corrected for. The use of *T. gondii *and *T. spiralis *antigens on two bead levels in a combined bead-based test to determine the serological status of swine would provide a new innovative assay which could be used as an alternative to ELISA in a *T. gondii *and *T. spiralis *monitoring system.

In this report, the specifications of a bead-based array test, with combined *T. gondii *and *T. spiralis *antigen bead levels to determine specific antibodies in serum of experimentally infected swine, are evaluated and compared to commercial and non-commercial ELISAs.

## Methods

### Porcine sera

#### Experimental infection sera

Swine serum samples originated from an experimental co-infection of pigs with *T. gondii *and *T. spiralis *[8]. Before infection, animals used in the experiment were assumed *T. gondii *and *T. spiralis *free on basis of the post partum determined negative serological status of sows which gave birth to these animals [8]. Briefly, eight to nine week old animals had been singly (*T. gondii *n = 8, *T. spiralis *n = 10), simultaneously (n = 10), or successively (*T. gondii*/*T. spiralis *n = 9, *T. spiralis*/*T. gondii *n = 10) orally inoculated with either 2,700 or 2,000 *T. gondii *tissue cysts (strain DX) and/or 5,000 *T. spiralis *muscle larvae (strain ISS 14) per pig. Because two animals of the *T. spiralis*/*T. gondii *inoculated group did not seem to be infected, they were excluded from the experiment [8]. A total of 444 serum samples were collected in series at 0, 5, 12, 19, 26, 33, 40, 47 and 54 days post infection (p.i.) from 45 pigs and four additional non-inoculated animals which served as negative control animals. This animal study, under number DEC 2008.III.03.023, was reviewed and approved by the local animal ethics committee according to the recommendations of the EU directive 86/609/EEC. Numbers of animals and their suffering were minimized.

#### Negative field sera

Blood samples of conventional finisher pigs were collected for *Salmonella *baseline monitoring at the abattoir in 2007 by the Dutch Food and Consumer Product Safety Authority (nVWA). The blood was left at room temperature with a minimum of 2 hours to clot and subsequently centrifuged for 10 minutes at 1,100 × g. Serum was drawn and dispensed in aliquots and kept at -20°C until further use.

Serum samples were analyzed by a commercially obtained *T. gondii *ELISA (ID Screen Toxoplasmosis Indirect, ID-VET, Montpellier, France; hereafter referred as E3-TOX). Serum samples remaining under the designated cut-off value of the ELISA were considered to originate from *T. gondii *infection negative pigs. Because during the sample period no pigs with *T. spiralis *infections were reported [9], all animals were considered *T. spiralis *infection negative.

### Indirect assays

Sera from the experimentally infected animals were tested by the bead-based assay for *T. gondii *and *T. spiralis *antibodies simultaneously (hereafter referred to as BBA-TOX and BBA-TRI, respectively), by an RIVM in-house *T. gondii *ELISA (hereafter referred to as E1-TOX) and two commercially available *T. gondii *ELISA kits (Safepath, Carlsbath, CA, USA, hereafter referred to as E2-TOX) and E3-TOX, and by one *T. spiralis *ELISA (Safepath, hereafter referred to as E-TRI). All *T. gondii *indirect tests used an antigen based on *T. gondii *tachyzoites, of which E3-TOX utilized a recombinant tachyzoite surface protein (SAG-1) as antigen. The *T. spiralis *tests were based on ES antigens. Bead-based assays were run according to the specifications described in the section bead-based assay. Testing with the in-house ELISA [3] was described earlier [8] and included an intra-plate correction of E1-TOX data. All commercial ELISAs were run according to the specification of the kit providers. For E3-TOX, normalization of data was included.

### Bead-based assay

A bead-based assay was developed for simultaneous detection of specific antibodies which were captured by *T. spiralis *and *T. gondii *antigens on two different bead levels. NSB was recorded with reference beads, which is a bead level without coupled antigens. Each bead level was recognized via the emission of light with a unique intensity and wavelength of the beads intrinsic fluorescence. Specific and NSB in each individual serum sample were determined by the extrinsic response, which was generated by the emission of light by a fluorophore attached to the secondary antibody. Because NSB may vary between serum samples, the extrinsic response of reference beads was used to determine the non-specific response. To obtain a specific response per individual serum sample, this non-specific response was subtracted from the response of coupled beads. A BD Accuri flow cytometer was used for enumeration of micro-particles, excitation of fluorescent markers and measurement of emitted light from these markers.

#### Chemicals, materials and solutions

L4, L10 and L11 carboxylated Cyto-plex™ beads (cat# FM5CR04, FM5CR10 and FM5CR11, respectively) were purchased from Thermo Scientific (Waltham, MA, USA). *T. gondii *tachyzoite lysate, strain RH (cat#: R29123) was from Meridian Life Science Inc. (Saco, ME, USA). *T. spiralis *Excretory/Secretory antigen (ES) was obtained from Instituto Superiore Sanità (Rome, Italy). Microcentrifuge copolymer tubes (cat# 1415-2500) were acquired from Star Lab GmbH (Ahrensberg, Germany). *N*-hydroxysulfosuccinimide sodium salt (sNHS), *N*-(3-dimethylaminopropyl)-*N*'-ethylcarbodiimide hydrochloride (EDC; cat#: 03449) and 2-(*N*-morpholino)ethanesulfonic acid hydrate (MES; cat#: M8250) were bought from Sigma-Aldrich Chemie B.V. (Zwijndrecht, the Netherlands). A 45 mM MES buffer was prepared and adjusted to pH 6.0 with sodium hydroxide. PBS at pH 7.2 consisted of 0.01 M sodium chloride (NaCl, Merck KGaA, Darmstadt, Germany), 1 mM di-sodium hydrogen phosphate (Merck) and 3 mM potassium dihydrogen phosphate (Merck). Water was of milliQ quality. Storage buffer and HNT-PBS solution were provided by RnAssays (Utrecht, the Netherlands). The 0.45 μm filter plates (cat#: MSHVN4550) were from Millipore (Amsterdam, the Netherlands). Goat anti-swine secondary antibody conjugated with fluorescent DyLight 488 was purchased from Jackson Immuno Research (West Grove, PA, USA).

#### Bead coupling procedure

*T. gondii *tachyzoite lysate and *T. spiralis *Excretory/Secretory antigen (ES) were coupled to carboxylated beads through an amine coupling procedure. Briefly, an equivalent of 1.4 × 10^8 ^carboxylated beads of L10 and L11 were transferred to two 1.5 ml copolymer tubes. The beads were washed by three repeats of following steps: a 3 minutes centrifugation at 9,000 ×*g*, removal of the supernatant, addition of 1 ml water per tube and resuspension of the beads on a vortex. After the third removal of supernatant, beads from both tubes were resuspended in 1.1 ml solution consisting of 12.5 mg sNHS and 12.5 mg EDC in MES buffer. This suspension was incubated for 20 minutes at room temperature on a gyro rocker at 70 rpm. Beads were washed 2 more times with 500 μl water as described above and after removal of the supernatant, 50 μg of *T. gondii *lysate and 10 μg *T. spiralis *ES dissolved in 200 μl PBS pH 7.4 were added to the activated L10 and L11 beads, respectively. Resuspended beads were left to incubate for 2 hours on a gyro rocker at 70 rpm, washed and stored in a storage buffer. A non-coupled L4 reference bead suspension was produced with the same protocol with exception of the protein incubation step which was substituted by PBS incubation. This L4 bead is referred to as the reference bead.

#### Assay procedure

Two 0.45 μm filter plates were soaked with 150 μl of a 0.2 μm filtered solution of HNT-PBS, subsequently incubated for five minutes at ambient temperature, and emptied by vacuum filtration. Serum samples were diluted 1:50 in HNT-PBS, transferred to a soaked and aspirated 0.45 μm filter plate, filtered with the use of the vacuum manifold and collected in an empty 96-wells plate. Thereafter, in another soaked filter plate, a quantity of approximately 5 × 10^5 ^*T. gondii *and *T. spiralis *antigen coupled beads and reference beads were suspended in 50 μl of HNT-PBS per well. Subsequently, one equivalent volume of filtered diluted sera was mixed and incubated with the bead-mix per well for 15 minutes on an orbital shaker (1,050 rpm). Beads were washed with 200 μl HNT-PBS by aspiration and additionally incubated with 100 μl 1:300 in HNT-PBS diluted fluorescent secondary antibody for 15 minutes. Finally, beads were washed once more and suspended in 100 μl HNT-PBS. Due to light sensitivity of beads and fluorescent reporter the filter plates were protected from light during incubation steps.

#### Internal and external fluorescent detection

A total of 600 beads per serum sample were analyzed for the intrinsic bead label on the FL4 channel, and extrinsic fluorescence reporter label on the FL1 channel using a BD Accuri C6 flow cytometer (BD Accuri Cytometers, Inc. Ann Arbor, MI, USA). The detector was equipped with a CSampler liquid handler (BD Accuri) and operated through CFlow software (version 1.0.243.1, BD Accuri). Beads were transported at a flow rate of 35 μl/min. The emission of the intrinsic fluorescence of the three bead levels, measured by the FL4 filter at 675 nm, was used to distinguish the *T. gondii *(TOX), *T. spiralis *(TRI) and reference (REF) beads. The median extrinsic fluorescence intensity (MFI) of the secondary antibody per bead level was determined by measuring the emission via the FL1 filter at 530 nm.

#### Correction for non-specific binding

Reference beads were used to indicate the measure of NSB in the test. Differences in NSB on uncoupled or antigen coupled bead levels may be expected due to differences in affinity of beads for non-specific antibodies caused by the molecular structure of the antigen, its orientation and concentration on the bead surface. Therefore, to estimate the NSB on *T. gondii *and *T. spiralis *bead levels from the response of a reference bead, a correction factor was calculated by testing 932 *T. gondii *and 13 extra *T. spiralis *negative swine sera (section Negative field sera) in the bead-based assay. With the use of least square regression, linear relations, expressed with the formulae y = slope*x + intercept, the relation between responses of the reference beads (x value) and *T. gondii *and *T. spiralis *bead responses (y values) were calculated in SPSS 16.0 for Windows (SPSS Inc., Chicago, IL, USA). Because the residuals of the linear relation between responses of *T. gondii *and *T. spiralis *bead levels and reference bead responses were not normally distributed, all responses were log transformed.

#### Normalization of responses

To compare results between 96-wells plates, serum samples responses were normalized. The percentage of normalized responses (%NR) was calculated as a percentage of sample responses (MFI^S^) of a positive control response (MFI^PC^), which was present in quadruplicate on each plate, after subtraction of NSB (sections Correction for non-specific binding and Results and Discussion).

(1) $$\%\text{N}{\text{R}}_{\text{T}}=\;{\left(\left(\text{MF}{\text{I}}^{\text{S}}-\text{ NS}{\text{B}}^{\text{S}}\right)/\left(\text{MF}{\text{I}}^{\text{PC}}-\text{ NS}{\text{B}}^{\text{PC}}\right)\right)}_{\text{T}}*\text{1}00\%$$

where subscript T represents *T. gondii *or *T. spiralis *in the considered case.

### Statistical analysis

All statistical evaluations were performed with SPSS.

To specify the performance of the bead-based assay, expressed in area under the curve (AUC), receiving operator characteristic (ROC) calculations were performed using the experimental infection status as gold standard. Analysis was performed on the %NR of the BBA and E3-TOX, and OD_450nm_of the other ELISAs. ROC calculations were also performed on limited sets of serum from the experimental infection. These sets consisted of all samples minus serum samples drawn 5 days after inoculation with *T. gondii *(n = 408) and all samples minus serum samples drawn 5, 12 and 19 days after inoculation with *T. spiralis *(n = 360), for the *T. gondii *and *T. spiralis *indirect tests, respectively. To further specify the tests, diagnostic Sensitivity (Se), Specificity (Sp) and cut-off values at maximum Youden Index were determined from ROC calculations [10].

To assess the agreement between tests, the marginal homogeneity of paired proportions [11] were tested by McNemar's in a 2 × 2 contingency table. Furthermore, inter-rater agreement was calculated using Cohen's Kappa. For this, serum responses of all tests were labelled 0 (negative), when they were below the cut-off value or 1 (positive) when they were equal or above cut-off value. These dichotomized outcomes where then evaluated against the dichotomized outcomes of the other tests. Kappa values between 0.40 - 0.59, 0.60 - 0.79 and ≥ 0.80 are interpreted as moderate, substantial and excellent agreement, respectively [11].

The apparent prevalence (AP) is the proportion of the population which tests positive in the test, which is a measure of true prevalence (TP) and the capability of the test to predict true positives and negatives, and it was calculated as [12]:

(2) $$\text{AP}=\text{Se }*\text{ TP}+\left(\text{1}-\text{Sp}\right)\;*\left(\text{1}-\text{TP}\right)$$

where TP is the proportion of actual infected animals which was calculated by:

(3) TP=n/N

where n is the number of sera which originate from inoculated animals, and N is the total number of sera.

## Results and discussion

Results from *T. gondii *and *T. spiralis *negative field samples, negative control serum of the experimental infection and secondary antibody binding alone (Table 1) showed that the height of NSB responses is foremost dependent of the presence of serum. Observing that negative sera due to NSB can reach the same or higher responses as for example positive control serum of 250,000 MFI on *T. gondii *and 500,000 MFI on *T. spiralis *beads (data not presented), it is concluded that correction for NSB is necessary to prevent false positive results.

**Table 1:** Non specific binding responses of *T.gondii *and *T. spiralis *negative serum sets and conjugate alone

Serum	Bead identification	n	Min. response (MFI)	Max. response (MFI)	Mean response (MFI)	SE
Buffer	REF	98	300	600	500	5
	
	TOX	98	3,900	4,700	4,300	19
	
	TRI	98	3,200	3,900	3,500	17

(*T. gondii *or *T. spiralis*) negative field sera	REF	947	400	491,500	53,700	2,200
	
	TOX	932	4,200	269,200	47,900	1,100
	
	TRI	945	3,400	505,500	75,300	2,300

Experimental infection negative control sera	REF	36	1,100	1,900	4,100	600
	
	TOX	36	4,900	45,500	21,500	1,900
	
	TRI	36	4,200	34,800	10,400	1,000

Minimum, maximum and mean response signals of reference (REF), *T. gondii *(TOX) and *T. spiralis *(TRI) coupled beads after incubation of buffer, negative field sera (without positive responders in *T. gondii *ELISA) and sera of the negative control animals of the experimental infection. n, number of tested sera; SE, standard error

**Figure 1:**
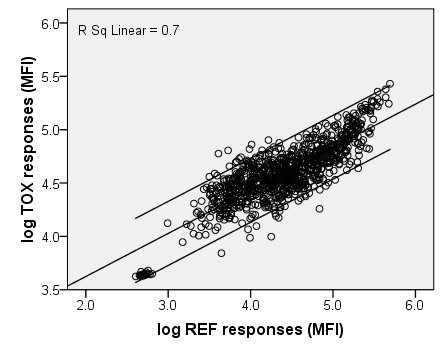
**Estimation of *T. gondii *non-specific binding correction factor**. Log transformed responses of *T. gondii *negative swine field sera on non-coupled beads (x-axis) versus *T. gondii *coupled beads (y-axis). Linear regression line (*log *y = 0.404* *log *x + 2.818), 95%CI lines and the linear R^2 ^are presented.

**Figure 2:**
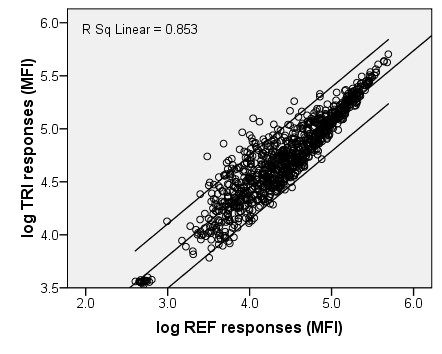
**Estimation of *T. spiralis *non-specific binding correction factor**. Log transformed responses of *T. spiralis *negative swine field sera on non-coupled beads (x-axis) versus *T. spiralis *coupled beads (y-axis). Linear regression line (*log *y = 0.646*log x + 1.863), 95%CI lines and the linear R^2 ^are presented

The results of *T. gondii *and *T. spiralis *negative field sera illustrate that the response of the reference beads could not directly be used as a measure for non-specific binding. The *log *linear relations between responses of reference beads and *T. gondii *or *T. spiralis *beads are depicted in Figures 1 and 2, and the relations were expressed as:

(4) $$log\text{NS}{\text{B}}_{\left(\text{TOX}\right)}=log\text{MF}{{\text{I}}_{\text{REF}}}^{*}0.\text{4}0\text{4}+\text{2}.\text{818}$$

(5) $$log\text{NS}{\text{B}}_{\left(\text{TRI}\right)}=log\text{MF}{{\text{I}}_{\text{REF}}}^{*}0.\text{646}+\text{1}.\text{863}$$

The slopes of the two regression lines indicate that *T. gondii *beads are subject to less NSB as compared to *T. spiralis *beads. This finding may be explained by, for example, variable concentration of antigens on the bead, differences in antigen molecule structures and orientation on the bead surface and/or the affinity between non-specific antibodies and the unoccupied bead surface or coupled antigens. A variety in antigen composition between biologically produced batches can therefore be of importance in relation to NSB. To test whether the correction factor to calculate bead correlated NSB is stable between batches of antigen, further evaluation is necessary. Subtraction of uncorrected responses of reference beads, according to formula 1, would lead to an underestimation or overestimation of NSB for responses below and above 53,480 and 183,110 for *T. gondii *and *T. spiralis*, respectively.

Results of ROC calculations, presented in Table 2, showed that the AUC, a measure of agreement between specific responses and the experimental infection status of the animals, of all indirect tests ranged between 0.837 and 0.930 for *T. gondii *and 0.855 and 0.879 for *T. spiralis*. These values indicate that there is a good relation between the responses of all indirect tests and the infection status of the animals.

**Table 2:** Infection status based specifications of bead-based assay and ELISA tests calculated by ROC analysis

	ROC analysis						
					**Maximum Youden Index**		

**test**	**AUC**	**SE**	***P***	**95% CI**	**Cut-off**	**Se**	**Sp**

BBA-TOX	0.911	0.015	< 0.001	0.881-0.940	13.90	0.86	0.96

E1-TOX	0.837	0.018	< 0.001	0.800-0.873	0.369	0.64	0.95

E2-TOX	0.865	0.018	< 0.001	0.829-0.901	0.174	0.76	0.93

E3-TOX	0.930	0.013	< 0.001	0.904-0.956	26.90	0.84	0.99

BBA-TRI	0.855	0.018	< 0.001	0.819-0.890	4.65	0.68	1.00

E-TRI	0.879	0.016	< 0.001	0.849-0.912	0.018	0.72	0.95

ROC analyses of a *T. gondii *and *T. spiralis *bead-based assay, three *T. gondii *and one *T. spiralis *ELISAs using responses of porcine serum samples from an experimental infection calculated against their infection status. AUC, area under the curve; SE, standard error of AUC; *P*, P-value; 95% CI, interval at a 95% confidence; Se, sensitivity; Sp, specificity; BBA-TOX, *T. gondii *antigen coupled bead-based assay; E1-TOX, RIVM in-house *T. gondii *ELISA, E2-TOX: Safepath *T. gondii *ELISA, E3-TOX, ID-VET *T. gondii *ELISA; BBA-TRI, *T. spiralis *antigen coupled bead-based assay and E-TRI, Safepath *T. spiralis *ELISA

**Figure 3:**
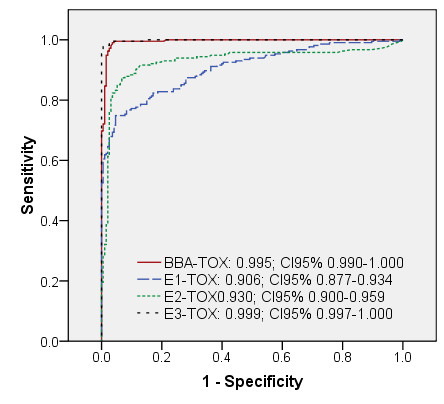
**ROC curves of *T. gondii *assays calculated with a limited serum set from experimental infection**. ROC analysis of a *T. gondii *bead-based assay and three ELISAs using responses from a set of serum samples, consisting of sera from experimentally infected pigs minus sera drawn on day 5 after inoculation with *T. gondii*, against their infection status. AUC, area under the curve; 95%CI, interval of AUC at 95% confidence; BBA-TOX, *T. gondii *antigen coupled bead-based assay; E1-TOX, RIVM in-house *T. gondii *ELISA, E2-TOX: Safepath *T. gondii *ELISA, E3-TOX, ID-VET *T. gondii *ELISA.

**Figure 4:**
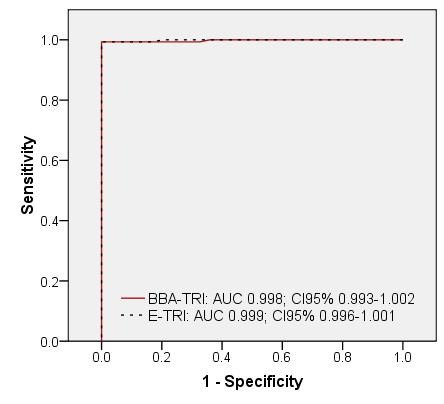
**ROC curves of *T. spiralis *assays calculated with a limited serum set from experimental infection**. ROC analysis of a *T. spiralis *bead-based assay and ELISA using responses from a set of serum samples, consisting of sera from experimentally infected pigs minus sera drawn on days 5, 12 and 19 after inoculation with *T. spiralis*, against their infection status. AUC, area under the curve; 95%CI, interval of AUC at 95% confidence; BBA-TRI, *T. spiralis *antigen coupled bead-based assay; E-TRI: Safepath *T. gondii *ELISA.

A perfect test is a test in which the responses correspond 100%, i.e. an AUC of 1.000, with the values of the test to which it is compared. The imperfect AUC values (< 1.000) found in our study (Table 3) can partly be explained by a late immunological development of antibodies, which is associated to the time course of parasite antigen expression and the immune response of infected animals. Evidence from earlier studies showed that muscle larvae, depending on the infection dose, can be found in pork by digestion or trichinoscopy as early as 17 days p.i. [13]. Other studies showed that *T. spiralis *ES could be measured within the developing muscle larvae and its cuticular surface as early as 14 days p.i. [14] and in the surrounding tissue around 15 days p.i. [15]. Consequently, the response time, i.e. the time of development of antibodies against the antigen used in the indirect *T. spiralis *assays, is affected by this late production. Porcine IgG antibodies against ES are developed approximately 3 to 4 weeks after infection with 5,000 muscle larvae [16,17]. Porcine IgG antibodies against *T. gondii *tachyzoites are produced much earlier in time and can be detectable after one to two weeks of infection [18]. In our study, the sera used for ROC calculations originated from animals which were collected on a weekly basis [8]. Samples drawn 5 days after *T. gondii *inoculation and 5, 12 and 19 days after *T. spiralis *inoculation would produce a false negative result when compared to the experimental infection status, resulting in lower AUC values. Calculations of ROC curves without these sera resulted in notably higher AUC values of 0.995, 0.999, 0.998 and 0.999 for BBA-TOX, E3-TOX, BBA-TRI and E-TRI, respectively (Figures 3 and 4).

**Table 3:** Inter-test agreement between assays calculated by McNemar's and Cohen's Kappa analysis

	McNemar's test Yates correction	Cohen's Kappa	
**Test comparison**	***P***	κ *	**95% CI**

BBA-TOX vs. E1-TOX	< 0.001	0.676	0.607-0.744

BBA-TOX vs. E2-TOX	0.01	0.802	0.746-0.857

BBA-TOX vs. E3-TOX	0.06	0.932	0.900-0.966

E1-TOX vs. E2-TOX	< 0.001	0.752	0.690-0.814

E1-TOX vs. E3-TOX	< 0.001	0.723	0.658-0.787

E2-TOX vs. E3-TOX	0.233	0.797	0.740-0.853

BBA-TRI vs. E-TRI	< 0.001	0.880	0.835-0.925

Inter-test agreement calculations comparing between dichotomized results of tests. *P*, probability; *κ*, Cohen's Kappa value; 95% CI, interval at a 95% confidence; BBA-TOX, *T. gondii *antigen coupled bead-based assay; E1-TOX, RIVM in-house *T. gondii *ELISA; E2-TOX, Safepath *T. gondii *ELISA; E3-TOX, ID-VET *T. gondii *ELISA; BBA-TRI, *T. spiralis *antigen coupled bead-based assay and E-TRI, Safepath *T. spiralis *ELISA. * indicates that all values were statistically significant (P < 0.001)

According to data of test performances and inter test agreement, presented in Table 3, the *T. gondii *and *T. spiralis *bead assays agree excellently with their respective highest scoring tests, i.e. with the E-TOX3 and E-TRI, respectively. Marginal homogeneity by McNemars test, a test which determines the equality between positive and negative test proportions of one test compared to the other, indicated that there is a balance between BBA-TOX and E-TOX3.

The potential use of the bead based test is prevention of *T. gondii *and/or *T. spiralis *infections in pork to enter the human food chain. Nonetheless, direct parasitological testing, like *T. spiralis *artificial digestion, are more reliable methods to test for present infection in meat. Existing *T. gondii *direct tests are either laborious, e.g. due to the need of pathogen extraction in PCR [19], or are undesirable because of utilization of experimental animals, like in cat and mice bioassays [20], to determine the infectious status of meat. In the case of *T. gondii *infections, serological testing is the next best option to perform on large scale. Unfortunately, due to the time window between infection and development of specific antibody responses, serological tests are less reliable for detection on individual scale; however, they can be used for monitoring purposes on herd level [21].

To prevent human *T. gondii *and *T. spiralis *infections through consumption of infected pork by serological monitoring of pig herds, a high sensitivity of 99% [22], and an approximation of AP to TP (Table 4) is desired. None of the assays used for this paper met this requirement. However, when ROC calculations were restricted to serum samples in which antibody responses were to be expected, as was described above, the sensitivity was 97% and 99% for BBA-TOX and BBA-TRI, respectively (data not presented). Subsequent calculations for true and apparent prevalence resulted in an overall *T. gondii *TP of 52.7% and AP's of 51.5% and 53.3% for BBA and ELISA, respectively. The overall TP of *T. spiralis *was 44.4% while the APs were 44.1% and 44.3% for BBA and ELISA, respectively (data not presented). These data would indicate that the combined bead-based assay is applicable for serological monitoring purposes.

**Table 4:** Comparison of true and apparent prevalence's of pig sera between indirect assays

test	True prevalence	Apparent prevalence
BBA-TOX	56.6%	50.1%
		
E1-TOX		38.3%
		
E2-TOX		45.9%
		
E3-TOX		48.0%

BBA-TRI	58.6%	39.8%
		
E-TRI		44.2%

BBA-TOX, *T. gondii *antigen coupled bead-based assay; E1-TOX, RIVM in-house *T. gondii *ELISA; E2-TOX, Safepath *T. gondii *ELISA; E3-TOX, ID-VET *T. gondii *ELISA; BBA-TRI, *T. spiralis *antigen coupled bead-based assay and E-TRI, Safepath *T. spiralis *ELISA

Although we compared our new *T. gondii *and *T. spiralis *bead test with a limited selection of available ELISAs, the test specifications and agreement between tests examined in this study indicate that the combined bead test equals or is superior to other tests. However, all calculations have been based upon tests using serum samples of experimentally infected pigs, which were exposed to high doses of parasites. Conventionally raised animals are likely to be infected by lower doses of parasites, and sero-conversion may be detected later in time [16-18]. Therefore, to determine the applicability of this bead-based test for the use of indirect detection of infection, it is advisable to further evaluate the test by the use of serum samples of naturally infected pigs.

## Conclusions

In conclusion, this initial evaluation study of a novel bead-based assay capable of a simultaneous detection of serological antibodies against *T. gondii *and *T. spiralis *antigens indicates that the test results correspond very well to the infection status of the animals, and, furthermore, there is a substantial to excellent agreement with other indirect tests. In order to estimate the applicability of this test for purposes of serological monitoring, further testing of sera from naturally infected animals is required.

## Authors' contributions

GCAMB helped in conceiving of the study, developed and optimized the bead-based test method, analyzed serological material in the test methods, statistically interpreted the data and wrote the manuscript. AAB and FvK both conceived of the study, participated in its design and coordination, contributed their expertise and helped to draft the manuscript. All authors read and approved the final manuscript.
